# Supplementary material for: One-step generation of T-cell receptor knock-in mice in the TCRβ locus
Source: EMBO J. 2026 May 13;45(12):4321–36. doi: 10.1038/s44318-026-00779-z (PMC13269491; doi:10.1038/s44318-026-00779-z)
Supplement: Supplementary file 1 — Appendix [file 44318_2026_779_MOESM1_ESM.pdf]

Appendix for *One-step generation of TCR knock-in mice targeted to the TCRβ locus results in functional mature T lymphocytes*

Table Of Contents

**Appendix figure title**	**Page**
Appendix Figure S1. TCRβ and CD5 expression among escapees.	1
Appendix Figure S2. Frequency of γδ, CD4 and CD8 T cells in spleens of TCR KI mice.	1
Appendix Figure S3. Nur77 expression in peripheral T cells.	2
Appendix Figure S4. *In vitro* polarization of TCR KI T cells.	2
Appendix Figure S5. Bcl-6 expression in Tfh cells after protein immunization and PR8 infection.	3

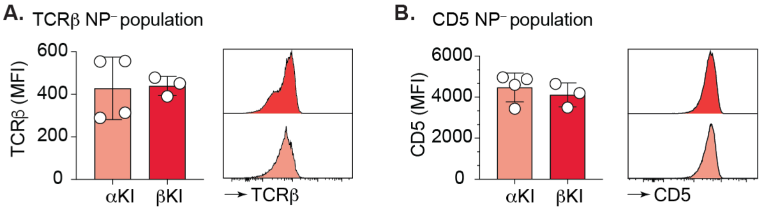

Appendix Figure S1 (related to Figure 2). TCRβ and CD5 expression among escapees. Graphs and histograms show the expression of TCRβ (A) and CD5 (B) among NP-tetramer$^-$ CD4$^+$ thymocytes from the indicated TCR KI mice. Data are pooled from three experiments with n=3-4 biological replicates. For all plots data and error bars represent the mean ± s.e.m.

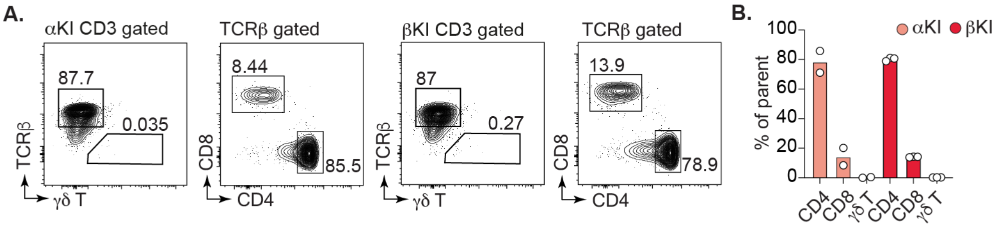

Appendix Figure S2 (related to Figure 3). Frequency of γδ, CD4 and CD8 T cells in spleens of TCR KI mice. A) Representative flow cytometry plots indicating gating strategy for γδ CD8 and CD4 T cells. B) Quantification of major T cell subsets (γδ out of CD3$^+$ T cells and CD4$^+$ or CD8$^+$ out of TCRβ$^+$ T cells). Data show one experiment with n=2-3 biological replicates. For all plots data and error bars represent the mean ± s.e.m.

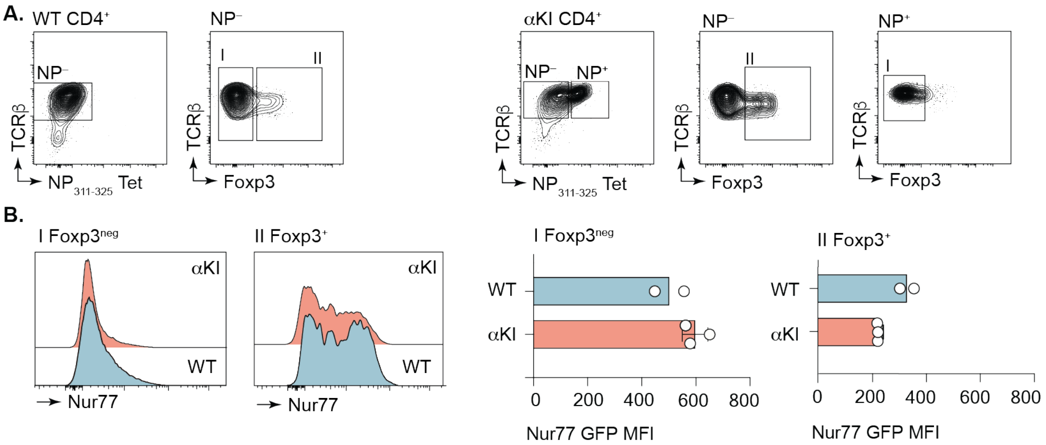

Appendix Figure S3. Nur77 expression in peripheral T cells. A) Representative plots showing Foxp3 expression in NP+ and NP− populations in WT and αKI TCR mice crossed to Nur77-GFP reporter mice. B) Bar graphs show MFI for the Nur77 GFP fluorescence for NP-tetramer+ or NP-tetramer− KI T cells and MFI of CD4+ WT T cells. Data show one experiment with n=2-3 biological replicates. For all plots data and error bars represent the mean ± s.e.m.

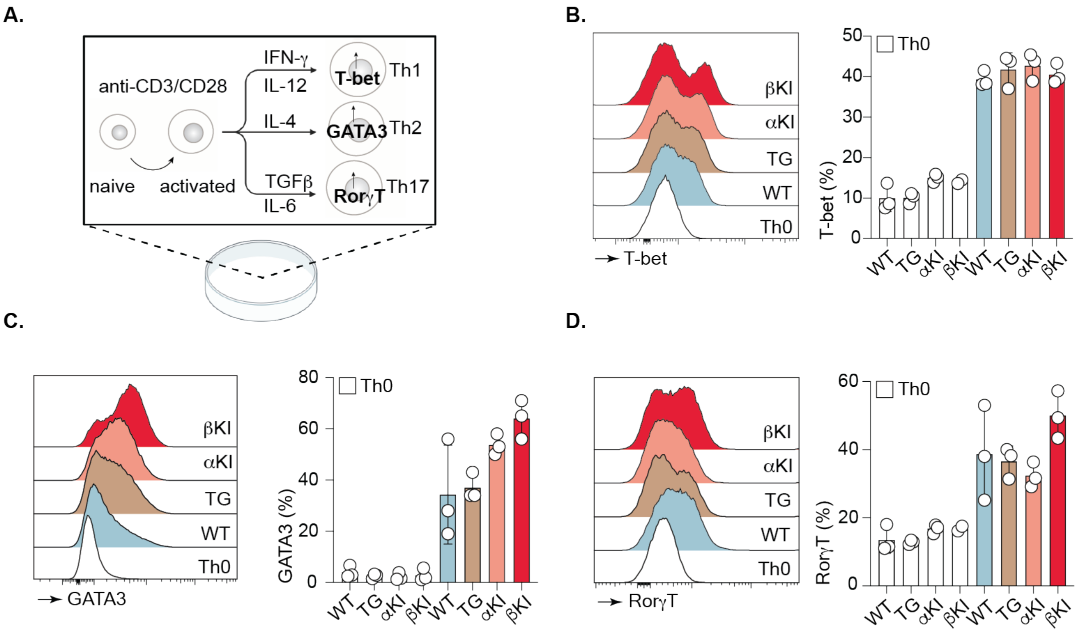

Appendix Figure S4. *In vitro* polarization of TCR KI T cells. A) Schematic showing *in vitro* polarization assay with stimulating and differentiating factors to obtain Th1, Th2 and Th17 with their respective transcription factors that were stained for and measured. Polarization of WT, TG, αKI and βKI T cells into Th1 B), Th2 C), and Th17 D) is summarized as mean ± s.e.m for T-bet, GATA3 and RorγT positive T cells respectively. Representative histograms are shown compared to Th0 for each condition. For all plots data and error bars represent the mean ± s.e.m. Data show one experiment with n=3 technical replicates.

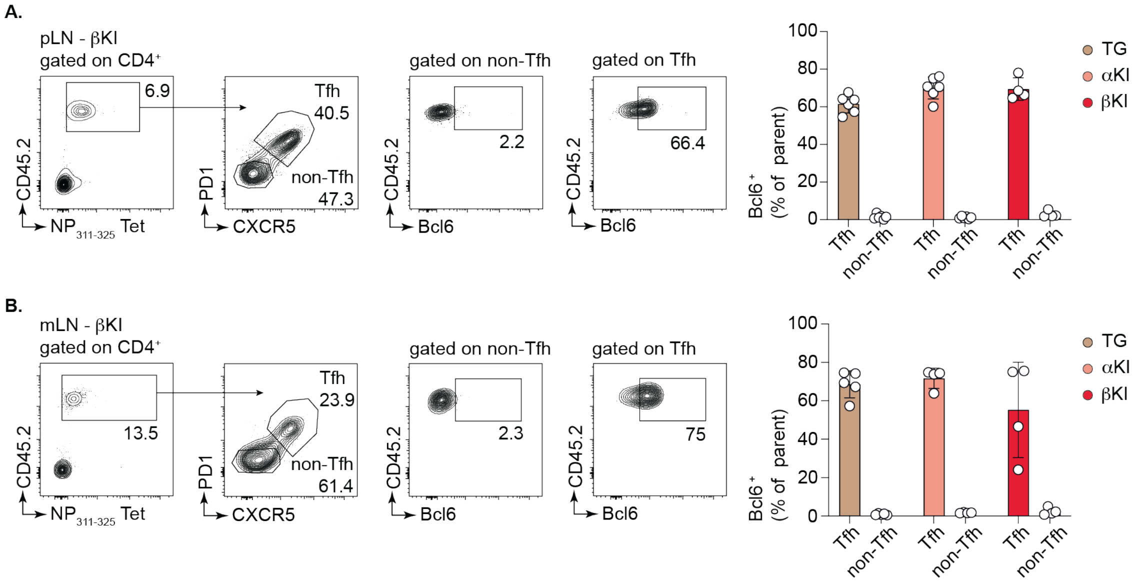

Appendix Figure S5 (related to Figure 4). Bcl-6 expression in Tfh cells after protein immunization and PR8 infection. A) Representative flow cytometry plots from the pLN of a protein-immunized mouse showing gating strategy for Bcl6 expression gated on Tfh and non-Tfh of transferred $CD4^{+}$ NP^{+} $CD45.2^{+}$ T cells. Graph shows quantification. B) Representative flow cytometry plots from the mLN of a PR8-infected mouse showing gating strategy for Bcl6 expression gated on Tfh and non-Tfh of transferred $CD4^{+}$ NP^{+} $CD45.2^{+}$ T cells. Graph shows quantification. Data are pooled from two experiments with n=4-6 biological replicates. Data and error bars represent the mean ± s.e.m. pLN, popliteal lymph nodes; mLN, mediastinal lymph nodes.
