# Supplementary material for: One-step generation of T-cell receptor knock-in mice in the TCRβ locus
Source: EMBO J. 2026 May 13;45(12):4321–36. doi: 10.1038/s44318-026-00779-z (PMC13269491; doi:10.1038/s44318-026-00779-z)
Supplement: Supplementary file 2 — Peer Review File [file 44318_2026_779_MOESM2_ESM.pdf]

One-step generation of T-cell receptor knock-in mice in the TCRβ locus

Jana Bilanovic, Juliana Bortolatto, Susu Duan, Valgerdur Bjørnsdottir, A. Katharina Teetz, Alexandra Thoms, Julian Fishbach, Fabian Schmidt, William Nyberg, Harald Hartweger, Amelia Escolano, Paul Thomas, Gabriel Victora, Angelina Bilate, and Johanne Jacobsen

Corresponding author(s): Angelina Bilate (abilate@rockefeller.edu) , Johanne Jacobsen (j.t.jacobsen@medisin.uio.no)

Review Timeline:

Submission Date:	24th Apr 25
Editorial Decision:	26th Jun 25
Revision Received:	5th Jan 26
Editorial Decision:	13th Feb 26
Revision Received:	10th Mar 26
Accepted:	18th Mar 26

Editor: Ioannis Papaioannou

Transaction Report:

(Note: With the exception of the correction of typographical or spelling errors that could be a source of ambiguity, letters and reports are not edited. Depending on transfer agreements, referee reports obtained elsewhere may or may not be included in this compilation. Referee reports are anonymous unless the Referee chooses to sign their reports.)

Dear Angelina, dear Johanne Tracey,

Thank you again for submitting your manuscript EMBOJ-2025-121127 for consideration by The EMBO Journal, and for your patience during peer review. As I have already informed you, your manuscript has now been seen by three experts in the field, and we have received their detailed and informative reports that I have already shared with you (they are included again below).

I am pleased to say that the referees find the method described in your work significant, relevant, and useful, and mention that the manuscript is detailed and well-written. While they are largely supportive of the work, they also identify a number of limitations and raise a few concerns, providing detailed suggestions for strengthening the work and the manuscript further. We think that the reports are balanced, fair, and constructive, and are glad to hear that you also find them constructive and the points raised largely addressable.

Given the referees' supportive comments and positive recommendations, as well as your feedback, I would like to invite you to submit a revised version of your manuscript taking the referees' suggestions on board, along with a detailed point-by-point response addressing all referees' comments. I should add that it is The EMBO Journal policy to allow only a single round of major revision, and acceptance of your manuscript will therefore depend on the completeness of your responses in this revised version. As we have already discussed, I kindly encourage you to share with me a brief point-by-point response to the referees' comments already during the initial stages of your revision, commenting particularly on any points you do not agree with or cannot address during the revision, to discuss how to best proceed. Please also feel free to contact me if you have any questions or other comments you would like to discuss with me.

We generally allow three months as standard revision time (September 25, 2025). As a matter of policy, competing manuscripts published during this period will not negatively impact our assessment of the conceptual advance presented by your study. However, we request that you contact us as soon as possible upon publication of any related work, to discuss how to proceed. Should you foresee a problem in meeting this three-month deadline, please let us know in advance and we will be able to grant an extension.

Thank you for the opportunity to consider your work for publication in The EMBO Journal. I look forward to your revision.

Best regards,

Ioannis

Ioannis Papaioannou, PhD
Editor, The EMBO Journal
i.papaioannou@embojournal.org

*****
Instructions for preparing your revised manuscript
*****

1. When you are ready to submit the revision, please upload:

- A Word file of the manuscript text (including legends of main Figures, EV Figures and Tables). Please make sure that changes are highlighted (or "tracked") to be clearly visible.

- Individual production-quality figure files (one file per figure). When assembling your figures, please refer to our figure preparation guidelines in order to ensure proper formatting and readability in print as well as on screen:
https://bit.ly/EMBOPressFigurePreparationGuideline
If the data shown in a figure are obtained from n {less than or equal to} 2, please use scatter plots showing the individual data points.
Please also include scale bars in all microscopy images.
The following points must be specified in each figure legend:
i. the name of the statistical test used to generate error bars and P values
ii. the number (n) of independent experiments (please specify technical or biological replicates) underlying each data point (discussion of statistical methodology can be reported in the Materials and Methods section, but figure legends should contain a basic description of n, P, and the test applied)
iii. the nature of the bars and error bars (s.d., s.e.m.).
See also guidelines for figure legends: https://www.embopress.org/page/journal/14602075/authorguide#figureformat

- A point-by-point response to the referees' comments, with a detailed description of the changes made (as a word file). All referees' concerns must be fully addressed and their suggestions taken on board. When preparing your letter of response to the referees' comments, please bear in mind that this will form part of the Review Process File and will therefore be available online to the community. Please note that you have the possibility to opt out of the transparent process at any stage prior to publication by letting the editorial office know (contact@embojournal.org); if you do opt out, the Review Process File link will point to the following statement: "No Review Process File is available with this article, as the authors have chosen not to make the review process public in this case.". For more details on our Transparent Editorial Process, please visit our website: https://www.embopress.org/page/journal/14602075/authorguide#transparentprocess

- Expanded View (EV) files (replacing Supplementary Information) that are collapsible/expandable online. A maximum of 5 EV Figures can be typeset. EV Figures should be cited as "Figure EV1, Figure EV2" etc. in the text, and their respective legends should be included in the manuscript file after the legends of regular figures. See detailed instructions regarding Expanded View files here:
https://www.embopress.org/page/journal/14602075/authorguide#expandedview

- For the figures that you do NOT wish to display as Expanded View figures, they should be bundled together with their legends in a single PDF file called "Appendix", which should start with a short Table of Contents (including page numbers). Appendix figures should be referred to in the main text as: "Appendix Figure S1, Appendix Figure S2" etc. Please see detailed instructions here: https://www.embopress.org/page/journal/14602075/authorguide#expandedview

- A complete author checklist, which you can download from our author guidelines (https://www.embopress.org/page/journal/14602075/authorguide). Please note that the checklist will also be part of the Review Process File.

2. Please note that no statistics should be calculated and shown in Figures if n=2. Please also note that each p value should be reported as an exact value.

3. Before submitting your revision, primary datasets (and computer code, where appropriate) produced in this study need to be deposited in appropriate public databases (see https://www.embopress.org/page/journal/14602075/authorguide#dataavailability). The accession numbers, database, and the specific URLs (links) should be listed in a formal "Data availability" section (placed after Methods), following the example below:

"The RNA-seq datasets produced in this study are available in the following database:
Gene Expression Omnibus GSE46843 (https://www.ncbi.nlm.nih.gov/geo/query/acc.cgi?acc=GSE46843)"

*** All links should resolve to a page where the data can be accessed. ***

*** Please remember to provide in the Data availability section of your revised manuscript reviewer passwords if the datasets are not yet public. ***

*** The Data Availability Section is restricted to new primary data that are part of this study. In case you have no data that require deposition in a public database, please state so instead of referring to the database: "Our study includes no data deposited in public repositories." under the heading "Data availability". ***

4. The materials and methods need to be described in the manuscript using our structured methods format, which is now required for all research articles. According to this format, the Methods section includes a single "Reagents and Tools Table" - listing key reagents, experimental models, software and relevant equipment including their sources and relevant identifiers- followed by a "Methods and Protocols" section describing the methods. Please download and fill our Reagents and Tools Table template (.docx), which you can find in our author guide:
https://www.embopress.org/page/journal/14602075/authorguide#structuredmethods. When submitting your revised manuscript, please do not include the Reagents and Tools Table in the Methods section of the manuscript but instead upload it as a separate file choosing the file type "Reagent Table".

5. Please check that the title and the abstract of the manuscript are brief, yet explicit, even to non-specialists. The length of the title should not exceed 100 characters, and the abstract should be a single paragraph not exceeding 175 words.

6. Please also note our reference format: https://www.embopress.org/page/journal/14602075/authorguide#referencesformat.

7. At EMBO Press we ask authors to provide source data for the main manuscript figures. You will receive a separate email with instructions for providing source data with your revised manuscript, including how to upload and organize the files.

8. Please remember: digital image enhancement is acceptable practice, as long as it accurately represents the original data and conforms to community standards. If a figure has been subjected to significant electronic manipulation, this must be noted in the

figure legend or in the "Materials and Methods" section. The editors reserve the right to request original versions of figures and the original images that were used to assemble the figure.

9. Our journal encourages inclusion of data citations in the reference list to directly cite datasets that were obtained from public databases. Data citations in the article text are distinct from normal bibliographical citations and should directly link to the database records from which the data can be accessed. In the main text, data citations are formatted as follows: "Data ref: Smith et al, 2001" or "Data ref: NCBI Sequence Read Archive PRJNA342805, 2017". In the Reference list, data citations must be labeled with "[DATASET]". A data reference must provide the database name, accession number/identifiers, and a resolvable link to the landing page from which the data can be accessed at the end of the reference. Further instructions are available at: https://www.embopress.org/page/journal/14602075/authorguide#referencesformat.

10. We request authors to consider both actual and perceived competing interests. Please review our policy (https://www.embopress.org/page/journal/14602075/authorguide#conflictsofinterest) and update your competing interests statement if necessary. Please name this section 'Disclosure and competing interests statement' and place it after the Acknowledgements section.

11. Please note that all corresponding authors are required to provide an ORCID ID upon submission of a revised manuscript (https://orcid.org/). Please find instructions on how to link your ORCID ID to your account in our manuscript tracking system in our Author guidelines (https://www.embopress.org/page/journal/14602075/authorguide#authorshipguidelines).

12. We use CRediT to specify the contributions of each author in the journal submission system. CRediT replaces the author contribution section, which should be removed from the manuscript. Please use the free text box to provide more detailed descriptions. See also guide to authors: https://www.embopress.org/page/journal/14602075/authorguide#authorshipguidelines.

13. Further information is available in our Guide For Authors: https://www.embopress.org/page/journal/14602075/authorguide

14. We would also welcome the submission of cover suggestions or motifs to be used by our Graphics Illustrator in designing a cover.

15. Please use the link below to submit your revision:
https://emboj.msubmit.net/cgi-bin/main.plex

Referee #1:

The manuscript by Bilanovic et al. describes a very efficient generation of a knock-in mouse for the α/β T cell receptor using AAV and CRISPR/Cas9 technology in mouse zygotes. The technology described is (surprisingly) the first-of-its-kind and therefore essential to be published. The authors correctly stated that transgenic technology used for many TCRs causes various problems, which are addressed by the "new" technology. The technology, including detailed technical information, is very well described. The results describe all the essential analyses one would like to have to appreciate the physiologic expression of the TCR as well as the functions of the T cells that develop in these mice. An interesting effect on T cell differentiation is also described after influenza infection, which, although not essential for the novelty and importance of the manuscript, is worth noting.

Here are my minor comments in random order:
- As it appears from Figure 3B, just 62% of T cells bind to the tetramer as a clearly distinguishable population. This is in conflict to the notion that allelic exclusion is almost complete, as it is convincingly shown by the Vβ analysis in Fig. 3 F. This needs at least a discussion. Maybe the authors can provide data on gating for tetramer-negative T cells and further analysis for Vbeta chain expression by FACS. It would not be surprising (and not really a problem) that those T cells escaped allelic exclusion and expanded in the periphery. The choices for Vß rearrangement are plenty, as the second allele is intact and actually, the DJCβ1 locus is also intact. A careful reanalysis and/or discussion would resolve a certain degree of incomprehension among experts in thymus development and allelic exclusion.
- Fig. 3 E and F could profit from either reorganisation or clearer labeling. It isn't easy to understand, especially with the short accompanying text in the results section. Values for βKI are difficult to understand, given that only 38% of T cells are tetramer positive. The labeling for the schematic of the signaling apparatus must be g) in the legend
- Also for Fig. 3: Why not showing a TCRα/TCRβ FACS staining (on CD3 pregated cells) for the knock-in chains?
- The reference Brady et al. should be replaced by an earlier publication showing RAG downregulation PMID 8608942

- in the light of the mentioning of the-first-of-its-kind character of the knock-in approach for T cell receptor chains, it should be mentioned that a fully functional δ TCR chain was knocked in at the correct side in the locus and showed correct developmental expression, before PMID 37182210
- The FACS staining of wildtype mouse thymus for CD4 and CD8 is grossly deviating from "normal" percentages in the literature for DN (usually 3-5%) and SP (7-10% for CD4 and 5-8% for CD8. This should be reanalysed.

Referee #2:

The authors clearly outlined the history, utility, and pitfalls of transgenic TCR mice in research. With the advancements of CRISPR/Cas9 technology, it is logical to use this approach to improve on monoclonal TCR generation methods. Rollins, et al made knock-in TCR mice by targeting the abTCR to the Tra locus, which leads to more physiological thymocyte development, but requires endogenous rearrangement of the TCRb. This subsequently results in the concern of dual TCRb expression which could confound results. Here, Bilanovic et al. targeted the Trb locus with CRISPR/Cas9 and AAV6 to knock in abTCR from an NP-specific CD4+ T cell and generated two mouse strains, each utilizing a different promoter. The resulting T cells had accelerated thymocyte development due to premature TCR expression, but the authors showed that there was efficient allelic exclusion of the other Trb locus. This provides an additional tool for researchers to generate monoclonal TCR mice, depending on the needs for the project.
Overall, the authors very clearly outline their methods and show the KI TCR cells differentiate into different T helper subsets in vitro and in vivo. Comparison of the KI TCR CD4+ T cells to a TG CD4+ T cell with the same specificity but different TCR sequence does not seem to be the best comparison, especially when speculating about results being due to TCR affinity when no affinity measurements were taken (lines 265-268). I don't want to propose that the authors need to make a traditional TG mouse with their selected TCR sequence, or that they must make new KI TCR mice with the old TG TCR, but perhaps comparing the ratio of tetramer MFI:TCRb MFI of naïve NP+ T cells from the three groups could add more information about the relative avidities of the TCRs.
The manuscript could be strengthened with more information about the 30% of T cells that do not bind tetramer in the KI TCR mice, and perhaps re-analysis of the T cell subsets in the immunization and influenza infection experiments:
Approximately 70% of the KI TCR CD4+ T cells bind tetramer but the authors do not expound on this result. Is this due to poor allelic exclusion of the KI Trb or Tra locus and rearrangement of the endogenous loci? The TCR the authors chose utilizes TRBV3, which I believe is also known as Vb16. Vb16 was not used in Figure 3 F when characterizing the TCRb usage, I think because there is no commercially available antibody. If so, the authors could explicitly state this to explain why they are relying on tetramer binding to identify cells expressing the KI TCR. If the 30% of CD4+ cells that do not bind tetramer have an endogenously rearranged TCR, the Va2 and TCRb usage should look like the WT CD4+ T cells in Fig 3 E. The authors should show these data. Additionally, if the endogenous locus rearranges in some mice, there should be some CD8+ abT cells as well as gdT cells in these KI mice, which should be absent in a traditional TG T cell mouse. Please show this data as well. Finally, if KI mice are crossed to generate homozygous KI mice, does the percent of NP+ in CD4+ T cells jump to 100% in KI mice?
In some retrogenic approaches where the TCRa and TCRb chains are linked by p2A, the surface level of TCR is lower than in WT mice. Please show surface staining of the TCR, for example with H57.597 of the aKI, bKI, Tg, and WT mice, both for the thymus in Figure 2 and for peripheral tissues in Figure 3.
In vaccination and infection experiments with transferred KI TCR cells, in Fig 4 D, F and G, the data are presented as the percent of Tfh that are CD45.2 (Transferred TG or KI cells). What percent of TG or KI TCR CD4+ T cells become Tfh (rather than showing % of Tfh that are TG/KI)? The proportion of transferred cells in each subset could be influenced by the relative ability of the cells to expand in vivo. Additionally, in Fig 4 H the cells are pre-gated on TG/KI TCR cells and displayed as the % Tbet+ within CXCR5- PD1- gate. There are more Tfh in the KI TCR populations, so plotting % Tbet this way is inflating proportions a bit. It would be more accurate to present these data as % Tbet+ of all TG / TCR KI CD4+.
Minor notes:
• Fig 2 F - the slash in the Y axis label (CD24neg/TCRb+) was confusing - it implies it's a ratio
• The methods section and Fig 1 A state the mice were infected with the X31 strain of IAV, however the results section refers to PR8 (line 117). If it indeed was X31, the body of the text should be corrected.
• Typo on line 250: "the latter were been shown" should be "the latter were shown" or "the latter have been shown"
• Typo line 267: "differences in the affinities of each TCR to for pMHC" remove "to"
• Line 466: embryo is missing "e"

Referee #3:

General remarks:
In the manuscript titled "One-step generation of TCR knock-in mice targeted to the TCRβ locus results in functional mature T Cells", Bilanovic et al. describe the generation of TCR-transgenic mice through AAV-mediated CRISPR editing, inserting the transgene in the endogenous gene locus. The manuscript is well written, and the data are clearly visualized. The study is the second study describing TCR knock-in mice with orthotopic insertion, but in contrast to Rollins et al. the authors target the beta locus to prevent dual TCR beta chain occurrence. The described approach is highly relevant for immunological research in

mouse models. However, the interpretation of biological results is compromised by the lack of suitable control mice expressing the same TCR in a non-physiological setting.

Major points:
It is nice that the authors developed a mouse model with a novel TCR (which first had to be experimentally identified). However, a significant downside of this choice is that the results from the TCR knock-in mice cannot be compared to conventional transgenic mice. Orthotopic knock-in of, e.g., the SMARTA or the OT-II TCR would have enabled actual benchmarking against conventional transgenic TCR regulation (arguably with more constitutive TCR gene expression through extrinsic gene promoters).
Using such model TCRs would also have made it easier to analyze the behavior of TCR-knock-in T cells upon chronic antigen exposure (in chronic viral infection or tumor models), which represent settings in which the unique benefit of physiological TCR regulation might have become particularly apparent (since T cells may be better able to protect themselves from exhaustion through TCR downregulation). This would have enabled the authors to test whether the in-vivo phenotypes described by Eyquem et al., Nature 2017 (https://doi.org/10.1038/nature21405, this study should also be cited) in xenograft models would hold true with a knock-in TCR in a syngeneic setting.
While it would be clearly beyond the scope of the manuscript to generate another knock-in mouse line with a model CD4 TCR, the authors should be able to generate retrogenic mice for the influenza TCR, and thereby use a constitutively active gene promotor according to the conventional protocol described by Holst et al., Nat Protocols 2006 using viral transduction of hematopoietic stem cells (https://doi.org/10.1038/nprot.2006.61; this study should also be cited). The alternative transgenic TCR that is specific for the same influenza epitope that is not introduced until Fig. S2 is not suitable in this regard since it is a different TCR. Retrogenic mice with the same TCR would allow a more comparative baseline assessment of TCR expression levels, CD5 expression levels, etc. that the authors performed in the first figures and of the in-vivo reactivity shown in Fig. 4 (when the authors could only compare the knock-in TCR to polyclonal WT TCRs or the alternative transgenic TCR).
The authors could then also engineer recombinant viruses leading to chronic infection (like LCMV) to present the influenza epitope that their TCR is targeting, and/or use a tumor model generated in a similar manner. In any case, an in-vitro "stress/exhaustion test" should be possible to conduct, with repetitive antigen challenge over several days. This functional comparison of the knock-in mice to TCR transgenics underlying artificial TCR regulation would significantly strengthen the manuscript. While such a revision is admittedly somewhat extensive, it appears feasible given the authors' expertise.

Minor points:
The study by Hahn et al., Cell Rep 2023 (https://doi.org/10.1016/j.celrep.2023.112253), should be cited, which represents - to my knowledge - the first description of generation of a mouse model with orthotopic TCR insertion, albeit in their case for the γδ TCR. Roth et al., Nature 2018 (https://doi.org/10.1038/s41586-018-0326-5), should also be cited as the first study to show orthotopic TCR knock-in in primary T cells (then human), as well as Schober et al., Nat Biomed Eng 2019 (https://doi.org/10.1038/s41551-019-0409-0), because this study demonstrated the importance of dual chain editing in the setting of TCR knock-in.
In the abstract and at the end of the introduction, it should be mentioned that this study used a CD4 TCR for knock-in to aid the reader.
Fig. 1F: It would also aid the reader if, in the text accompanying this sub-figure, it would be explained that a certain leakiness of endogenous TCR expression is expected, and the degree of leakiness should be interpreted in the context of the unique setting of this knock-in model (comparing the results, for example, to conventional TCR-transgenic mice with or without a RAG1/2 KO background).
Fig. 2H: Please provide exemplary primary data for Fig. 2H (CD5 levels), best in the form of vertically aligned histograms, so that the CD5 expression levels of the monoclonal TCR can be compared to the expression levels of the polyclonal WT population (like later in Fig. 3). For both TCR expression levels and CD5, it would also be nice to see the coefficient of variation of transgenic TCRs vs. polyclonal WT TCRs (the same applies to Fig. 3H-L). For analyses like these, it would also be informative to show (in a supplementary figure) the NP tetramer-negative population of knock-in mice as an internal negative control.
Fig. 3B: Please comment on the potential nature of the CD44- CD62L- population in Q3, which appears even more distinct in the knock-in mice than in WT mice.
Fig. 3D-F: First, please remind the reader about the TRAV/TRBV configuration of the knock-in TCR (TRAV6-7/DV9/TRAJ57 for TCRa chain and TRBV3/TRBD2/TRBJ7 for TCRb chain according to the genetic IMGT nomenclature) and explain which alpha or beta chains this corresponds to in the Wilson nomenclature, which is usually used for v chain-targeting flow cytometry antibodies. This should be v beta 16 for the beta locus according to IMGT (https://www.imgt.org/IMGTrepertoire/index.php?section=LocusGenes&repertoire=nomenclatures&species=mouse&group=TRBV), which is not covered by the shown antibody stainings. This important information is so-far missing. Second, it took me a while to see that in Fig. 3F the y-axis has a different scale, which should be highlighted or differently visualized to make the important point that leaky polyclonal TRAV/TRBV expression is much lower in the knock-in mice than in WT mice. The y-axis should also be changed to "Vα/Vβ(x) expression..." to be more accurate and make the x-axis better comprehendible. The color legend on top is not horizontally aligned. Third, paired alpha-beta TCR repertoire sequencing would add significantly to this part of the manuscript, since it would not only validate correct expression of the desired TCR in knock-in mice, but also enable a more comprehensive assessment of the polyclonal background expression of other TCRs genes. This aspect appears particularly important given that the engineering aspect of the manuscript at hand represents an advance beyond the study of Rollins et al., Nat Comm 2023, mostly because the endogenous beta chain is targeted.
The helpful brief speculation of the authors why the v beta promotor knock-in mice did not show leakiness for endogenous beta

chains should appear earlier in the text (when Fig. 3F is introduced) since otherwise the reader is left wondering why this could be the case until the end of the text's paragraph is reached.
The figure legend of Fig. 3 is wrongly annotated from Fig. 3G onwards.
Figure 4: As outlined in the major comments above, these analyses would considerably benefit from a retrogenic control of the same TCR that was used for the knock-in TCR. With the present data at hand, it is hard to interpret the differences to the alternative transgenic TCR since TCR avidity differences may mask regulation-effects, as openly discussed by the authors themselves. In-vitro testing of peptide sensitivity would also be an interesting readout in this context (both to see differences in TCR regulation when the same TCR is used through, e.g., knock-in and retrogenic mice, and to see differences between the knock-in TCR and the alternative TCR to validate the assumed avidity differences - it is not clear to me from which source the authors take their confidence in the differential avidity given that the TCR used for the knock-in mice was newly identified in the present study).
In the discussion, the role of TCR affinity remains unclear given the limitations of same-TCR comparisons (as outlined above). The insinuated superiority of the presented workflow to the approach by Rollins et al. - although it is intuitive - should also be phrased more carefully because experimental evidence for less heterogeneity and less confounding affects with the system of the manuscript at hand could not be sufficiently shown. The discussion should also include a paragraph that addresses the question how physiological the approach with inserted v alpha or v beta promotors is compared to natural gene regulation. In this regard, it should be an interesting food for thought for the reader that in natural polyclonal populations each TCR that has a different V segment underlies differential gene regulation.

We thank all the reviewers for their thorough evaluation of our manuscript and helpful suggestions. We have addressed the concerns raised by the reviewers in the revised version, and we believe the manuscript is much improved. All text changes in the new version of the manuscript are now marked in blue. We have also edited the text and figures to clarify our main message which is the development of a streamlined approach to generate TCR monoclonal knock-in mice. Please see our point-by-point responses below.

Referee #1:

The manuscript by Bilanovic et al. describes a very efficient generation of a knock-in mouse for the α/β T cell receptor using AAV and CRISPR/Cas9 technology in mouse zygotes. The technology described is (surprisingly) the first-of-its-kind and therefore essential to be published. The authors correctly stated that transgenic technology used for many TCRs causes various problems, which are addressed by the "new" technology. The technology, including detailed technical information, is very well described. The results describe all the essential analyses one would like to have to appreciate the physiologic expression of the TCR as well as the functions of the T cells that develop in these mice. An interesting effect on T cell differentiation is also described after influenza infection, which, although not essential for the novelty and importance of the manuscript, is worth noting.

We thank the reviewer for this positive evaluation and addressed all the minor comments raised by the reviewer below.

Here are my minor comments in random order:

- As it appears from Figure 3B, just 62% of T cells bind to the tetramer as a clearly distinguishable population. This is in conflict to the notion that allelic exclusion is almost complete, as it is convincingly shown by the Vβ analysis in Fig. 3 F. This needs at least a discussion. Maybe the authors can provide data on gating for tetramer-negative T cells and further analysis for Vbeta chain expression by FACS. It would not be surprising (and not really a problem) that those T cells escaped allelic exclusion and expanded in the periphery. The choices for Vß rearrangement are plenty, as the second allele is intact and actually, the DJCβ1 locus is also intact. A careful reanalysis and/or discussion would resolve a certain degree of incomprehension among experts in thymus development and allelic exclusion.

We agree with the reviewer that our description of escape was poorly phrased. We have modified the text to indicate that allelic exclusion is indeed not 100%, adding new text to the results and discussion that clarifies our interpretation of the data in the new Fig. 3F.

As the reviewer points out, because of how the pre-rearranged TCRαβ was inserted into the *Trb* locus, escapees can still rearrange the other allele. Nevertheless, roughly 60-70% of $CD4^+$ T cells in the TCR KI mice bind to the NP tetramer. To assess the nature of these "escapees" (non-binders) we analyzed TCRα/β expression by FACS of blood T cells and by single-cell TCR sequencing (scTCRseq) of splenic T cells. The new Fig. 3E-F show the protein expression of non-KI TCRβs and of TCR Vα2 among NP-tetramer binders and non-binders. Among binders, the expression of non-KI Vs was close to zero. In contrast, a larger fraction of the non-tetramer binders expressed non-KI TCRs, indicating that allelic exclusion occurs to some extent, but it is not 100%. We have edited the text accordingly.

To complement this analysis, we performed scTCRseq of NP-tetramer binders and non-binders. Our data show that, whereas NP binders carry the correct TCR KI rearrangement and are thus monospecific, roughly half of non-binders carry other, unique non-KI CDR3 rearrangements (see new Fig. 3G), indicating that rearrangement and expression of non-KI alleles can lead to loss of tetramer binding, possibly by mispairing between KI and non-KI TCR chains.

- Fig. 3 E and F could profit from either reorganisation or clearer labeling. It isn't easy to understand, especially with the short accompanying text in the results section. Values for βKI are difficult to understand, given that only 38% of T cells are tetramer positive. The labeling for the schematic of the signaling apparatus must be g) in the legend

Thank you for this suggestion and we apologize for the confusion. We modified the figure to clearly indicate the antibodies used and discern Vα2 from all other Vβ stainings. Additionally, we have also included the Vβ staining for both binders and non-binders (see new Fig. 3F). We have also removed the schematic for TCR signaling and expanded the results section to aid the reader.

- Also for Fig. 3: Why not showing a TCRα/TCRβ FACS staining (on CD3 pregated cells) for the knock-in chains?

We unfortunately are unable to stain specifically for the TCRα/β of our knock-in because monoclonals are not available for these particular V-segments. This is why we need to use tetramer staining along with the “negative” staining for the Vα2 and pan-Vβ, which identifies escapees. We added an explanatory note to this end to the text describing Fig. 3.

- The reference Brady et al. should be replaced by an earlier publication showing RAG downregulation PMID 8608942

We thank the reviewer for providing the PMID and have replaced the citation.

- in the light of the mentioning of the-first-of-its-kind character of the knock-in approach for T cell receptor chains, it should be mentioned that a fully functional δ TCR chain was knocked in at the correct side in the locus and showed correct developmental expression, before PMID 37182210

We thank the reviewer for this comment and have added this to the introduction.

- The FACS staining of wildtype mouse thymus for CD4 and CD8 is grossly deviating from "normal" percentages in the literature for DN (usually 3-5%) and SP (7-10% for CD4 and 5-8% for CD8. This should be reanalysed.

Thank you for pointing this out. We have included more mice in this analysis and reanalyzed the data. We added a new representative FACS plot in Fig 2B to reflect this change.

Referee #2:

The authors clearly outlined the history, utility, and pitfalls of transgenic TCR mice in research. With the advancements of CRISPR/Cas9 technology, it is logical to use this approach to improve on monoclonal TCR generation methods. Rollins, et al made knock-in TCR mice by targeting the abTCR to the Tra locus, which leads to more physiological thymocyte development, but requires endogenous rearrangement of the TCRb. This subsequently results in the concern of dual TCRb expression which could confound results. Here, Bilanovic et al. targeted the Trb locus with CRISPR/Cas9 and AAV6 to knock in abTCR from an NP-specific CD4+ T cell and generated two mouse strains, each utilizing a different promoter. The resulting T cells had accelerated thymocyte development due to premature TCR expression, but the authors showed that there was efficient allelic exclusion of the other Trb locus. This provides an additional tool for researchers to generate monoclonal TCR mice, depending on the needs for the project.

We thank the reviewer for this positive evaluation and addressed all the comments raised by the reviewer below.

- Overall, the authors very clearly outline their methods and show the KI TCR cells differentiate into different T helper subsets in vitro and in vivo. Comparison of the KI TCR CD4+ T cells to a TG CD4+ T cell with the same specificity but different TCR sequence does not seem to be the best comparison, especially when speculating about results being due to TCR affinity when no affinity measurements were taken (lines 265-268). I don't want to propose that the authors need to make a traditional TG mouse with their selected TCR sequence, or that they must make new KI TCR mice with the old TG TCR, but perhaps comparing the ratio of tetramer MFI:TCRb MFI of naïve NP+ T cells from the three groups could add more information about the relative avidities of the TCRs.

The reviewer is correct to point out that we cannot directly compare the properties of two TCRs with different CDR3 rearrangements. Our goal in including the TCR transgenic was to broadly benchmark our new method against a more traditional approach, not to compare specifics, but we agree that our language did not make this clear. We have modified the text and figures to remove these direct comparisons between models. The new edits are indicated in blue in the text (please see new text edits regarding Fig. 4).

The manuscript could be strengthened with more information about the 30% of T cells that do not bind tetramer in the KI TCR mice, and perhaps re-analysis of the T cell subsets in the immunization and influenza infection experiments:

- Approximately 70% of the KI TCR CD4+ T cells bind tetramer but the authors do not expound on this result. Is this due to poor allelic exclusion of the KI Trb or Tra locus and rearrangement of the endogenous loci?

We agree with the reviewer that our description of escape was insufficient. As the reviewer points out, about 70% of CD4 T cells in the TCR KI mice bind to NP tetramer. To more thoroughly assess the nature of the non-KI "escapees" we analyzed TCRα/β expression by FACS of blood T cells and by single-cell TCR sequencing (scTCRseq) of splenic T cells. The new Fig. 3E-F shows the protein expression of non-KI TCRβs and of TCR Vα2 among NP-tetramer binders and non-binders. Among binders, the expression of non-KI Vs was close to zero. In contrast, a larger fraction of the non-tetramer binders expressed non-KI TCRs, indicating that allelic exclusion occurs to some extent, but it is not 100%.

To complement the FACS analysis, we performed scTCRseq of NP-tetramer binders and non-binders. Our data show that, whereas NP binders carry the correct TCR-KI rearrangement and are thus monospecific, roughly half of non-binders carry other, unique non-KI CDR3 rearrangements (see new Fig. 3G-H), indicating that rearrangement and expression of non-KI alleles can lead to loss of tetramer binding, possibly by mispairing between KI and non-KI TCR chains.

Additionally, we now show in the new Fig. 4C and G that donor-derived Tfh cells recovered after immunization or infection are all NP-tetramer binders.

- The TCR the authors chose utilizes TRBV3, which I believe is also known as Vb16. Vb16 was not used in Figure 3 F when characterizing the TCRb usage, I think because there is no commercially available antibody. If so, the authors could explicitly state this to explain why they are relying on tetramer binding to identify cells expressing the KI TCR. If the 30% of CD4+ cells that do not bind tetramer have an endogenously rearranged TCR, the Va2 and TCRb usage should look like the WT CD4+ T cells in Fig 3 E. The authors should show these data. Additionally, if the endogenous locus rearranges in some mice, there should be some CD8+ abT cells as well as gdT cells in these KI mice, which should be absent in a traditional TG T cell mouse. Please show this data as well. Finally, if KI mice are crossed to generate homozygous KI mice, does the percent of NP+ in CD4+ T cells jump to 100% in KI mice?

The text has been updated to clarify this point. Indeed, no commercially available antibody exists to stain our specific Vβ16 chain. We hope the new layout of figure 3F also clarifies this issue. We have also added new flow cytometry data for γδ and $CD8^+$ T cells proportions in TCR KI mice (see new Supplemental Fig. 2).

While we see about 70% of NP-tetramer binders in heterozygous mice one would expect that homozygous mice would have less escapees. We have not intercrossed the TCR KI mice to homozygosity, but we predict that this would indeed increase the proportion of NP-binders, as reported previously for classic transgenic models (PMID: 7520367).

- In some retrogenic approaches where the TCRa and TCRb chains are linked by p2A, the surface level of TCR is lower than in WT mice. Please show surface staining of the TCR, for example with H57.597 of the aKI, bKI, Tg, and WT mice, both for the thymus in Figure 2 and for peripheral tissues in Figure 3.

We now show this data in Figure 2G and 3K, and, for clarification, we have added overlaid histograms showing TCRβ expression in WT and TCR KI mice.

- In vaccination and infection experiments with transferred KI TCR cells, in Fig 4 D, F and G, the data are presented as the percent of Tfh that are CD45.2 (Transferred TG or KI cells). What percent of TG or KI TCR CD4+ T cells become Tfh (rather than showing % of Tfh that are TG/KI)? The proportion of transferred cells in each subset could be influenced by the relative ability of the cells to expand in vivo.

We have now also added the flow cytometry plots and bar graphs showing the frequency of Tfh out of all transferred cells to new Figure 4C and 4G.

- Additionally, in Fig 4 H the cells are pre-gated on TG/KI TCR cells and displayed as the % Tbet+ within CXCR5- PD1- gate. There are more Tfh in the KI TCR populations, so plotting % Tbet this way is inflating proportions a bit. It would be more accurate to present these data as % Tbet+ of all TG / TCR KI CD4+.

It was not our intention to inflate the proportion of Tbet-expressing cells but rather highlight the ability of the transferred cells to not only differentiate into Tfh but also into Th1 cells. We would like to clarify that we aimed to characterize the phenotypic identity of the non-Tfh compartment rather than the absolute yield of Th1 cells. Both TG and KI T cells can differentiate into Th1 cells in the non-Tfh compartment. Our gating strategy allowed us to characterize the fate of the transferred cells that did not differentiated into Tfh. Please see the **Reviewer Figure 1** below showing the frequency of Tbet-expressing cells among all transferred cells.

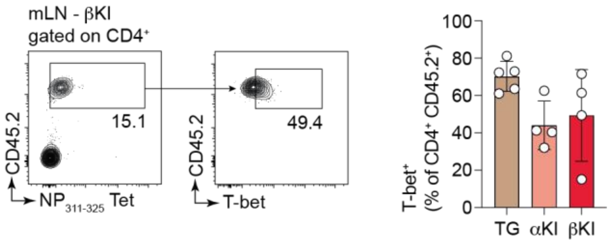

Reviewer Figure 1. Expression of T-bet among transferred TCR TG or TCR KI T cells. Representative flow cytometry plots showing T-bet expression on transferred $CD4^+$ NP^+ $CD45.2^+$ T cells. The plots are displaying the same representative sample as shown in Fig. 4H, now applying the T-bet gate on all transferred cells. Data are pooled from two independent experiments. Data and error bars represent the mean ± s.e.m.

Minor notes:

- Fig 2 F - the slash in the Y axis label (CD24neg/TCRb+) was confusing - it implies it's a ratio
- The methods section and Fig 1 A state the mice were infected with the X31 strain of IAV,

however the results section refers to PR8 (line 117). If it indeed was X31, the body of the text should be corrected.

• Typo on line 250: "the latter were been shown" should be "the latter were shown" or "the latter have been shown"

• Typo line 267: "differences in the affinities of each TCR to for pMHC" remove "to"

• Line 466: embryo is missing "e"

Thank you for noticing our mistakes. All of them have now been corrected.

<u>Referee #3:</u>

General remarks:

In the manuscript titled "One-step generation of TCR knock-in mice targeted to the TCRβ locus results in functional mature T Cells", Bilanovic et al. describe the generation of TCR-transgenic mice through AAV-mediated CRISPR editing, inserting the transgene in the endogenous gene locus. The manuscript is well written, and the data are clearly visualized. The study is the second study describing TCR knock-in mice with orthotopic insertion, but in contrast to Rollins et al. the authors target the beta locus to prevent dual TCR beta chain occurrence. The described approach is highly relevant for immunological research in mouse models. However, the interpretation of biological results is compromised by the lack of suitable control mice expressing the same TCR in a non-physiological setting.

We thank the reviewer for their positive comments and thorough reading of our manuscript. We believe the new data and text clarifies how we validated our approach to generate TCR KI mice.

Major points:

It is nice that the authors developed a mouse model with a novel TCR (which first had to be experimentally identified). However, a significant downside of this choice is that the results from the TCR knock-in mice cannot be compared to conventional transgenic mice. Orthotopic knock-in of, e.g., the SMARTA or the OT-II TCR would have enabled actual benchmarking against conventional transgenic TCR regulation (arguably with more constitutive TCR gene expression through extrinsic gene promoters).

Using such model TCRs would also have made it easier to analyze the behavior of TCR-knock-in T cells upon chronic antigen exposure (in chronic viral infection or tumor models), which represent settings in which the unique benefit of physiological TCR regulation might have become particularly apparent (since T cells may be better able to protect themselves from exhaustion through TCR downregulation). This would have enabled the authors to test whether the in-vivo phenotypes described by Eyquem et al., Nature 2017 (https://doi.org/10.1038/nature21405, this study should also be cited) in xenograft models would hold true with a knock-in TCR in a syngeneic setting. While it would be clearly beyond the scope of the manuscript to generate another knock-in mouse line with a model CD4 TCR, the authors should be able to generate retrogenic mice for the influenza TCR, and thereby use a constitutively active gene promotor according to the conventional protocol described by Holst et al., Nat Protocols 2006 using viral transduction of hematopoietic stem cells (https://doi.org/10.1038/nprot.2006.61; this study should also be cited). The alternative transgenic TCR that is specific for the same influenza epitope that is not introduced until Fig. S2 is not suitable in this regard since it is a different TCR. Retrogenic mice with the same TCR would allow a more comparative baseline assessment of TCR expression

levels, CD5 expression levels, etc. that the authors performed in the first figures and of the in-vivo reactivity shown in Fig. 4 (when the authors could only compare the knock-in TCR to polyclonal WT TCRs or the alternative transgenic TCR). The authors could then also engineer recombinant viruses leading to chronic infection (like LCMV) to present the influenza epitope that their TCR is targeting, and/or use a tumor model generated in a similar manner. In any case, an in-vitro "stress/exhaustion test" should be possible to conduct, with repetitive antigen challenge over several days. This functional comparison of the knock-in mice to TCR transgenics underlying artificial TCR regulation would significantly strengthen the manuscript. While such a revision is admittedly somewhat extensive, it appears feasible given the authors' expertise.

We agree with the reviewer that we cannot directly compare the current TG mice with our KI mice as they carry different CDR3 rearrangements, and we have removed the passages that made such comparisons in the revised version. Our intention with the TG mice was to provide a broad benchmark for our novel method in comparison with traditional approaches. Thus, we opted to generate mice using a TCR newly identified from the BALF of infected mice, which would be more useful to the field than making a second version of the same TCR already available. We have edited the text accordingly to clarify these points.

The primary goal of our effort was to create and validate an approach that uses CRISPR/Cas9 and AAVs to insert TCRs into an endogenous locus. The major advantage of these mice is that they are genetically defined, use endogenous regulatory elements, and are easier to generate than traditional TCR transgenics. While comparisons of the biology of T cells obtained with each approach would be interesting in principle, we would argue that any differences revealed by such comparisons would likely reflect problems with random and variable transgene integration in the transgenic or retrogenic, rather than with our targeted approach. For example, retrogenic mice can display lower TCR expression levels than traditional transgenic mice (PMID: 22348644) introducing other variables (TCR surface density) that would make it difficult to discern the effect of the expression system itself. We therefore believe that additional experiments with retrogenics (which differ from germline modified T cells in countless ways in addition to promoters and integration loci) to be beyond the scope of the present study.

Nevertheless, to investigate the question of endogenous vs. exogenous promoters, we expressed the same TCRα/β rearrangement used in our KI mice under the control of endogenous or strong exogenous promoters in mouse primary T cells using the Ark313 synthetic adeno-associated virus (PMID: 36638795). This method is a direct derivative of the one used by Eyquem et al. 2017 (PMID: 28225754) to target and express a CAR from the *Trac* locus. Thus, the TCRα promoter is the true endogenous control promoter in this case. In addition to the endogenous TCRα promoter, we chose a strong constitutive promoter commonly used for transgene expression, EF1α (PMID: 2210382), and the truncated weaker EFS promoter (PMID: 2210382). The latter is still considered a constitutive (housekeeping gene) promoter and is used for transgene expression (PMID: 39437851). This strategy allowed us to compare the impact of different promoters driving TCR expression in response to TCR engagement and restimulation *in vitro*. The experimental design is detailed in PMID: 36638795 and in the **Reviewer Figure 2** below. In short, primary T cells were isolated from Cas9-mice, activated with anti-CD3/CD28, and transduced with AAV donor vectors designed to insert our NP-specific TCRα/β cassette into the *Trac* locus. The TCR cassette was driven by one of the three different promoters mentioned above. Expression of TCR was then assessed after one or two rounds of TCR stimulation (see **Reviewer Figure 2**). Splenic TCR KI T cells expressing TCR under endogenous *Trac* promoter displayed downregulation of TCR upon anti-CD3/CD28 stimulation, which was not observed using a strong constitutive promoter. These results suggest that strong promoters may lock the T cells in an "on position," maintaining TCR expression upon restimulation, while under the endogenous *Trac* locus control, TCR expression is physiologically downregulated upon TCR restimulation (see **Reviewer Figure 2**). Since this is a very different system and not directly related to the *Trb* targeting approach

we used to generate germline TCR KI mice we opted for not showing this data in the manuscript. Nevertheless, these results highlight the utility of driving TCR expression through endogenous promoters.

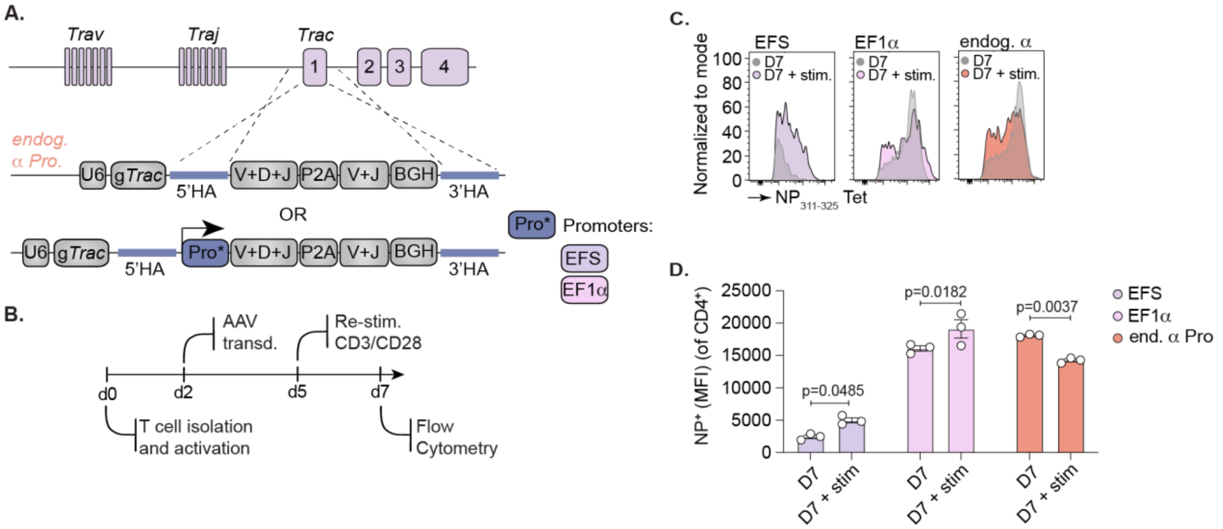

Reviewer Figure 2. Effect of promoter choice on TCR expression upon restimulation. A) Schematic representation of AAV template designs for *Trac* targeting. A template for NP-TCR expression from *Trac* was designed by using homology arms targeting exon 1 of *Trac* and a U6 promoter expressing a sgRNA targeting *Trac.* TCRβ and TCRα chains were separated by a P2A cleavable linker. Additional templates were designed to substitute *Trac*-regulation of the NP-TCR with inserted promoters, the NP-TCR sequences were followed by a stop codon and a BGH poly-A sequence. Two promoters were used for comparisons, EF1α and EFS. All templates were packaged into the Ark313 AAV serotype for T cell transductions. **B**) Splenic T cells were isolated from H11-Cas9 mice and activated using anti-CD3/CD28 dynabeads, followed by AAV transduction 48 hours later. Knock-in frequency was determined at D4 post-T cell activation. Half of the T cells were re-stimulated with an anti-CD3/CD28 dynabeads on D5 post initial T cell activation. Final flow cytometry analysis was performed on D7 post T cell isolation and activation. **C**) Representative histograms showing tetramer expression in $CD4^+$ T cells at D7 with or without re-stimulation. **D**) Graph shows the quantification of the MFI for tetramer on $CD4^+$ T cells D7 post T cell isolation and/or activation determined by flow cytometry. Tetramer expression was compared with- (D7 + stim.) or without re-stimulation (D7). Results are the mean ± SEM from three different cultures. Significance was assessed using Student's t test.

Minor points:

- The study by Hahn et al., Cell Rep 2023 (https://doi.org/10.1016/j.celrep.2023.112253), should be cited, which represents - to my knowledge - the first description of generation of a mouse model with orthotopic TCR insertion, albeit in their case for the γδ TCR. Roth et al., Nature 2018 (https://doi.org/10.1038/s41586-018-0326-5), should also be cited as the first study to show orthotopic TCR knock-in in primary T cells (then human), as well as Schober et al., Nat Biomed Eng 2019 (https://doi.org/10.1038/s41551-019-0409-0), because this study demonstrated the importance of dual chain editing in the setting of TCR knock-in.

These are relevant references that have now been added in context to the manuscript.

- In the abstract and at the end of the introduction, it should be mentioned that this study used a CD4 TCR for knock-in to aid the reader.

We have specified in the introduction that a $CD4^+$ T cell-derived TCR was used to generate the TCR KI mice.

- Fig. 1F: It would also aid the reader if, in the text accompanying this sub-figure, it would be explained that a certain leakiness of endogenous TCR expression is expected, and the degree of leakiness should be interpreted in the context of the unique setting of this knock-in model (comparing the results, for example, to conventional TCR-transgenic mice with or without a RAG1/2 KO background).

Thank you for this suggestion. In the revised manuscript, we added new text in the results and discussion sections to explain that endogenous TCR expression can occur in the TCR KI mice. In addition, to more thoroughly assess the nature of the non-KI "escapees" we analyzed TCRα/β expression by FACS of blood T cells and by single-cell TCR sequencing (scTCRseq) of splenic T cells. The new Fig. 3E-F shows the protein expression of non-KI TCRβs and of TCR Vα2 among NP-tetramer binders and non-binders. Among binders, the expression of non-KI Vs was close to zero. In contrast, a larger fraction of the non-tetramer binders expressed non-KI TCRs, indicating that allelic exclusion occurs to some extent, but it is not 100%.

To complement the FACS analysis, we performed scTCRseq of NP-tetramer binders and non-binders. Our new data showed that, whereas NP binders carry the correct TCR-KI rearrangement and are thus monospecific, roughly half of non-binders carry other, unique non-KI CDR3 rearrangements (see new Fig. 3G-H), indicating that rearrangement and expression of non-KI alleles can lead to loss of tetramer binding, possibly by mispairing between KI and non-KI TCR chains.

- Fig. 2H: Please provide exemplary primary data for Fig. 2H (CD5 levels), best in the form of vertically aligned histograms, so that the CD5 expression levels of the monoclonal TCR can be compared to the expression levels of the polyclonal WT population (like later in Fig. 3). For both TCR expression levels and CD5, it would also be nice to see the coefficient of variation of transgenic TCRs vs. polyclonal WT TCRs (the same applies to Fig. 3H-L). For analyses like these, it would also be informative to show (in a supplementary figure) the NP tetramer-negative population of knock-in mice as an internal negative control.

We have added these data as requested: Figure 2H now includes histograms of CD5 expression in WT and TCR KI T cells. New Supplemental Figure 1 shows the expression of TCRβ and CD5 among NP-tetramer^{-} T cells from TCR KI mice.

- Fig. 3B: Please comment on the potential nature of the CD44- CD62L- population in Q3, which appears even more distinct in the knock-in mice than in WT mice.

We speculate that this population which constitutes ~7-10% in WT and TCR KI mice may represent cells that have recently been activated or are undergoing homeostatic proliferation, leading to the transient downregulation of CD62L without full upregulation of CD44.

- Fig. 3D-F: First, please remind the reader about the TRAV/TRBV configuration of the knock-in TCR (TRAV6-7/DV9/TRAJ57 for TCRa chain and TRBV3/TRBD2/TRBJ7 for TCRb chain according to the genetic IMGT nomenclature) and explain which alpha or beta chains this corresponds to in the Wilson nomenclature, which is usually used for v chain-targeting flow cytometry antibodies. This should be v beta 16 for the beta locus according to IMGT (https://www.imgt.org/IMGTrepertoire/index.php?section=LocusGenes&repertoire=nomenclatures&species=mouse&group=TRBV), which is not covered by the shown antibody stainings. This important information is so-far missing. Second, it took me a while to see that in Fig. 3F the y-axis has a different scale, which should be highlighted or differently visualized to make the important point that leaky polyclonal TRAV/TRBV expression is much lower in the knock-in mice than in WT mice. The y-axis should also be changed to "Vα/Vβ(x) expression..." to be more accurate and make the x-axis better comprehendible. The color legend on top is not horizontally aligned. Third, paired alpha-beta TCR repertoire sequencing would add significantly to this part of the manuscript, since it would not only validate correct expression

of the desired TCR in knock-in mice, but also enable a more comprehensive assessment of the polyclonal background expression of other TCRs genes. This aspect appears particularly important given that the engineering aspect of the manuscript at hand represents an advance beyond the study of Rollins et al., Nat Comm 2023, mostly because the endogenous beta chain is targeted.

Thank you for raising these important points. We modified the text describing Figure 3 to clearly indicate the antibodies used and discern Vα2 from all other Vβ stainings. Additionally, we have also included the Vβ staining for both NP-tetramer binders and non-binders (see new Fig. 3F). We unfortunately are unable to stain specifically for the TCRα/β of our knock-in because monoclonals are not available for these particular V-segments. This is why we used tetramer staining along with the "negative" staining for the Vα2 and pan-Vβ, which identifies escapees. We added an explanatory note to this end to the text describing Fig. 3.

As for the suggestion of TCR repertoire sequencing, please see reply above for your comment about Fig 1F.

- The helpful brief speculation of the authors why the v beta promotor knock-in mice did not show leakiness for endogenous beta chains should appear earlier in the text (when Fig. 3F is introduced) since otherwise the reader is left wondering why this could be the case until the end of the text's paragraph is reached.

We have moved the explanation and our approach to evaluate the expression of endogenous chains to the beginning of the results section "Physiological peripheral development and efficient TCR allelic exclusion for KI T cells" before we introduce the data shown in Figure 3.

- The figure legend of Fig. 3 is wrongly annotated from Fig. 3G onwards.

We apologize for this oversight; the figure legend has now been corrected.

- Figure 4: As outlined in the major comments above, these analyses would considerably benefit from a retrogenic control of the same TCR that was used for the knock-in TCR. With the present data at hand, it is hard to interpret the differences to the alternative transgenic TCR since TCR avidity differences may mask regulation-effects, as openly discussed by the authors themselves. In-vitro testing of peptide sensitivity would also be an interesting readout in this context (both to see differences in TCR regulation when the same TCR is used through, e.g., knock-in and retrogenic mice, and to see differences between the knock-in TCR and the alternative TCR to validate the assumed avidity differences - it is not clear to me from which source the authors take their confidence in the differential avidity given that the TCR used for the knock-in mice was newly identified in the present study).

Please see our response to the reviewer's first point, above. We agree that we cannot directly compare the biology of our KI T cells to those of transgenic mice using a different TCR and have removed such comparisons from the revised manuscript. As we argue above, we believe comparing our T cells to retrogenics (which differ from germline modified T cells in countless ways in addition to promoters and integration loci) would be beyond the scope of the present manuscript.

- In the discussion, the role of TCR affinity remains unclear given the limitations of same-TCR comparisons (as outlined above). The insinuated superiority of the presented workflow to the approach by Rollins et al. - although it is intuitive - should also be phrased more carefully because experimental evidence for less heterogeneity and less confounding affects with the system of the manuscript at hand could not be sufficiently shown. The discussion should also include a paragraph that addresses the question how physiological the approach with inserted v alpha or v beta promotors is compared to natural gene regulation. In this regard, it should be

an interesting food for thought for the reader that in natural polyclonal populations each TCR that has a different V segment underlies differential gene regulation.

We agree with the reviewer and apologize for the confusion. We have revised and edited the text where we compare our approach to that of Rollins et al. (2023) and removed any comparisons regarding TCR affinity.

We acknowledge that in a natural polyclonal repertoire, each V segment is controlled by its own unique regulatory elements, and our model represents a simplification of this complexity. However, we argue that using a single endogenous promoter is a significant advance over the non-physiological, constitutive strong promoters used in prior random-integration models and represents a critical advance to recapitulate physiological TCR regulation. We have now included a discussion about how physiological the approach with inserted Vα or Vβ promoters is compared to natural gene regulation.

1st Revision - Editorial Decision 13th Feb 2026

Dear Angelina,

Thank you again for the submission of your revised manuscript (EMBOJ-2025-121127R) to The EMBO Journal for our consideration, and for your patience during re-review. Your manuscript has now been seen by the three original referees who had previously assessed the first version of the study, and we have received their comments, which are appended below.

As you will see, referees #1 and #2 are completely satisfied with the revision, recognize that their initially raised concerns have been sufficiently addressed in a strengthened version of the manuscript, and recommend publication without any other comments. Referee #3, on the other hand, points out that certain concerns/suggestions were not addressed, but would still be supportive of the manuscript should a more complete and balanced discussion of the study's limitations be included. This referee also identifies a few additional minor issues that can all be textually addressed/clarified.

In light of this input from the referees, I would like to invite you to submit a final version of your manuscript, textually addressing the remaining points of referee #3. I would like to clarify here that no new experimental data are requested. Please also upload a point-by-point response to the remaining referee comments with detailed explanations and a description of any changes to the manuscript.

From the editorial side, there are also a few changes we need you to make in the final version of the manuscript, before we can move forward with its acceptance for publication in The EMBO Journal:

- Please remove the Figures from the main manuscript file, these should only be uploaded as individual, high-resolution, Figure files. Only the Figure legends should remain at the end of the manuscript (below the References list).

- Please change heading "Materials and Methods" to "Methods".

- Please change heading "Conflict of interest" to "Disclosure and competing interests statement".

- The author contributions statement should be removed from the manuscript file. Instead, we use CRediT to specify the contributions of each author in the journal submission system. Please feel free to use the free text box to provide more detailed descriptions during submission. See also our guide to authors for more information: https://link.springer.com/partners/embo-press/editorial-policies#Authorship.

- We kindly request you include in your resubmission a complete author checklist, which you can download from our author guidelines (https://link.springer.com/journal/44318/submission-guidelines#cms-Revised-submissions). Please note that this checklist will also be part of the Peer Review file that will be published alongside your article.

- Please note that an ORCID ID is mandatory for each co-corresponding author. No ORCID ID has been provided (in our system) for your co-author Johanne T. Jacobsen yet.

- Please update the nomenclature and callouts for your "Data Set 1" to "Dataset EV1". Please also provide a caption in a separate sheet in the same Excel file.

- We noticed that you have uploaded supporting Figures. These can either be included in the manuscript as Expanded View (EV) Figures, or in an Appendix. Expanded View Figures are collapsible/expandable online and should be cited as "Figure EV1, Figure EV2" etc. in the text, while their respective legends should be included in the manuscript file after the legends of regular ("main") Figures. Alternatively, you can bundle together all Figures (and their legends) that you do not wish to display as Expanded View Figures in a single PDF file named "Appendix", which should contain on its title page the heading "Appendix for:", followed by a Table of Contents including page numbers for all listed items. Appendix Figures should be referred to in the Appendix and throughout the main manuscript as "Appendix Figure S1", Appendix Figure S2", etc. Each Appendix Figure should be accompanied by its legend in the same PDF file. More information can be found in our guide to authors: https://link.springer.com/journal/44318/submission-guidelines#cms-Expanded-View-data.

- Please note that EMBO press papers are accompanied online by:
A) a short (2 sentences) summary of the findings and their significance,
B) 2-5 short bullet points highlighting the key results, and
C) a synopsis image in .jpg or .png format that is exactly 550 pixels wide and 300-600 pixels high (the height is variable). Please note that all text needs to be legible at the final size.
Please upload this information along with your revised manuscript (the text for A and B should be provided in a separate Word file).

- Materials and methods need to be described in the manuscript using our structured methods format, which is now required for all articles. According to this format, the Methods section includes a single "Reagents and Tools Table" -listing key reagents,

experimental models, software and relevant equipment including their sources and relevant identifiers- followed by a "Methods and Protocols" section describing the methods. Please download and fill our Reagents and Tools Table template (.docx), which you can find in our author guide: https://link.springer.com/journal/44318/submission-guidelines#cms-Revised-submissions. When submitting your revised manuscript, please do not include the Reagents and Tools Table in the Methods section of the manuscript but instead upload it as a separate file choosing the file type "Reagent Table".

- Thank you for uploading the Source Data for your Figures. We noticed that the Source Data for Figure 3M might have been mislabeled (according to your Source Data checklist, Source Data for Figure 3L have been provided instead of 3M). We would be grateful if you could please double-check and correct the labeling and/or the Source Data checklist, as appropriate.

- Please acknowledge the use of Biorender.com for creating some schematics at the end of the Methods section. Please also make sure that you have an appropriate Biorender license.

- Our data editors have checked your Figures and their legends, and raised the following queries. Please address all points below completely in your revised manuscript (all changes should be highlighted or "tracked"):
1. Please provide the exact p-values in the legends of Figures 2C, D, F, I; 3C, L, M.
2. Please note that information related to "n" is missing in the legends of Figures S2 B, S3 B, S4 B-D; S5 A, B.
3. Please note that the error bars must be defined in the legends of Figures S2 B, S3 B.

- The manuscript sections need to be named and ordered as follows: Title page - Abstract - Introduction - Results - Discussion - Methods - Data Availability - Acknowledgements - Disclosure and Competing Interests Statement - References - Figure Legends - main Tables (if there are any) - Expanded View Figure Legends.

- Please also note that as part of the EMBO Press transparent editorial process, The EMBO Journal publishes online a Peer Review File along with each accepted manuscript. This File will be published in conjunction with your paper and will include the referee reports, your point-by-point responses and all pertinent correspondence relating to the manuscript. Your Author's Checklist will also be published at the end of the Peer Review File. Please let us know in case you want to remove any data or figures from your point-by-point responses before they are published as part of the Peer Review File. Retaining unpublished data in the Peer Review File means that these count as published and that the Peer Review File would need to be referenced in future publications. Please let the editorial office know in case you want to remove any data from this file (contact@embojournal.org).

We look forward to seeing a final version of your manuscript as soon as possible. Please let us know if you have any questions and use this link to submit your revision: https://emboj.msubmit.net/cgi-bin/main.plex.

Best regards,

Ioannis

Ioannis Papaioannou, PhD
Editor, The EMBO Journal
i.papaioannou@embojournal.org

Referee #1:

All reviewer queries have been adequately addressed in the revised manuscript. I congratulate the authors for this excellent study.

Referee #2:

The authors have revised the manuscript well in response to reviewers comments. The manuscript makes an important contribution to the literature.

Referee #3:

The authors chose not to address my major point on benchmarking their TCR transgenic model against retrogenic mice, which I proposed as a "compromise" in light of the fact that generating a new TCR transgenic model for a TCR for which conventional TCR transgenic models exist seemed outside the scope of a revision. While I am disappointed to see that the authors did not implement my suggestion after about nine months of revision (and apparently also did not breed mice to homozygosity in this timeframe - see response to reviewer #2), my former positive assessment also still stands that the model does provide a valuable resource for the scientific community. I could accept if the paper was to be published with the current set of data, but I would then wish for an open discussion on the limitations of the work in the discussion section. Irrespective of whether one considers the lack of consistent (i.e. with the same TCR) benchmarking against conventional TCR transgenic models a major limitation, a discussion on how TCR expression levels (and thus T cell fate after and during antigen exposure) may differ between i) conventional TCR transgenic models, ii) endogenous gene-targeted "physiological" TCR transgenic models and iii) retrogenic mice would be in my eyes highly beneficial for the discussion.

Other minor remaining points:
Fig. 1B. It is confusing that the "universal TCR targeting vector" contains the Trbv promotor while in the text only the Trav promotor is mentioned first, and in Fig. 1D and later in the text both Trav and Trbv promotors are mentioned.
Fig. 3G-H: I assume the color in the pie charts is supposed to indicate the "correct" KI TCR - this should be clearly described in the figure legend. It is interesting that also in the multimer non-binding population the frequency of the correct TCRs is relatively large. The authors speculate this could be due to competition/mispairing with the endogenous TCR, but do not actually pursue this hypothesis. They should have the single-cell sequencing data to test this concept. Also, is there anything about the non-correct TCRs that can be found that could be related to the engineering strategy or are those other TCRs seemingly completely randomly generated? Can the authors also comment on why they set the multimer gate so strictly (to the right)? I guess in cells with an intermediate multimer MFI the correctly paired TCR rate may not be 100%.

Referee #3:

The authors chose not to address my major point on benchmarking their TCR transgenic model against retrogenic mice, which I proposed as a "compromise" in light of the fact that generating a new TCR transgenic model for a TCR for which conventional TCR transgenic models exist seemed outside the scope of a revision. While I am disappointed to see that the authors did not implement my suggestion after about nine months of revision (and apparently also did not breed mice to homozygosity in this timeframe - see response to reviewer #2), my former positive assessment also still stands that the model does provide a valuable resource for the scientific community. I could accept if the paper was to be published with the current set of data, but I would then wish for an open discussion on the limitations of the work in the discussion section. Irrespective of whether one considers the lack of consistent (i.e. with the same TCR) benchmarking against conventional TCR transgenic models a major limitation, a discussion on how TCR expression levels (and thus T cell fate after and during antigen exposure) may differ between i) conventional TCR transgenic models, ii) endogenous gene-targeted "physiological" TCR transgenic models and iii) retrogenic mice would be in my eyes highly beneficial for the discussion.

We have now edited our discussion section to address these points (see new paragraph lines 359-370), and clarified the text in lines 333-334, pointing out the limitations of the TCR KI mice, in addition to the points already raised in lines 314-339.

Other minor remaining points:

Fig. 1B. It is confusing that the "universal TCR targeting vector" contains the Trbv promotor while in the text only the Trav promotor is mentioned first, and in Fig. 1D and later in the text both Trav and Trbv promotors are mentioned.

We thank the reviewer for pointing this out and have edited the figure.

Fig. 3G-H: I assume the color in the pie charts is supposed to indicate the "correct" KI TCR - this should be clearly described in the figure legend.

We agree with the reviewer that the color usage should be exlained in the figure legend. We have edited the figure legend.

It is interesting that also in the multimer non-binding population the frequency of the correct TCRs is relatively large. The authors speculate this could be due to competition/mispairing with the endogenous TCR, but do not actually pursue this hypothesis. They should have the single-cell sequencing data to test this concept. Also, is there anything about the non-correct TCRs that can be found that could be related to the engineering strategy or are those other TCRs seemingly completely randomly generated? Can the authors also comment on why they set the multimer gate so strictly (to the right)? I guess in cells with an intermediate multimer MFI the correctly paired TCR rate may not be 100%.

We analyzed our single cell TCR sequencing data to assess the pairing information among the non-binder escapees. The <u>Reviewer Figure</u> below shows that a large fraction of non-binders uses at least one correct chain from the knock-in but pairs with an endogenous chain, as expected. Regarding the gate, we decided to set the gates rather strictly to ensure the cells sorted properly reflected true binders and true non-binders to the best of our abilities.

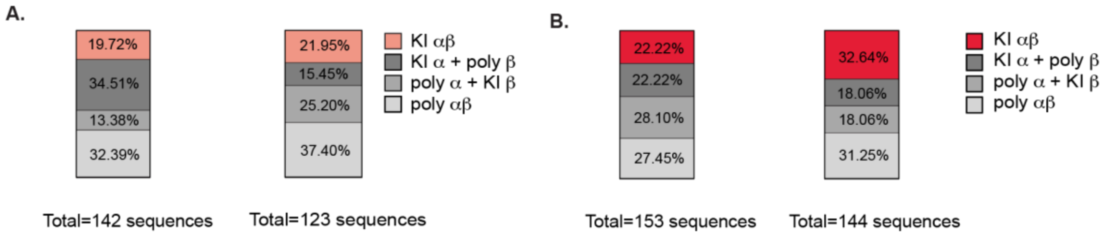

Reviewer Figure. Analysis of paired TCRαβ chains among tetramer^{-} T cells from TCR KI mice. scTCR sequencing analysis of splenic T cells from TCR KI mice. A) Pairing of TCRα and β chains in non-binders of the two αKI mice sequences as shown in Fig 3G. **B**) Pairing of TCRα and β chains in non-binders of the two βKI mice sequences as shown in Fig 3H. Colored slices indicate the proportion of T cells cells using the αβ pairing of the TCR KI mice. Gray slices indicate proportion of T cells expressing either the TCRα or TCRβ or endogenous rearrangements paired with an endogenous TCR chain as indicated. Poly, polyclonal endogenous rearrangements.

2nd Revision - Editorial Decision 18th Mar 2026

Dear Angelina,

Congratulations on an excellent manuscript! I am very pleased to inform you that it has been accepted for publication in The EMBO Journal. Thank you for addressing the initially raised referee concerns and our editorial requests for changes and corrections.

Your manuscript will be processed for publication by EMBO Press. It will be copy edited and you will receive page proofs prior to publication. Please note that you will be contacted by Springer Nature Author Services to complete licensing and payment information.

You may qualify for financial assistance for your publication charges - either via a Springer Nature fully open access agreement or an EMBO initiative. Check your eligibility: https://link.springer.com/journal/44318/how-to-publish-with-us

Should you be planning a Press Release on your article, please get in contact with embo_production@springernature.com as early as possible in order to coordinate publication and release dates.

If you have any questions, please do not hesitate to contact the Editorial Office. Thank you for your contribution to The EMBO Journal. Working with you has been a pleasure.

Yours sincerely,

Ioannis Papaioannou, PhD
Editor, The EMBO Journal
i.papaioannou@embojournal.org

*****

Please note that it is The EMBO Journal policy for the transcript of the editorial process (containing referee reports and your response letters) to be published as an online supplement to each paper. If you should prefer removal of any referee-only figures included in the point-by-point response(s), e.g. because they may still be used for future publication or because they have been reproduced from published work by others, please do let us know immediately via response email.
More information is available here: https://link.springer.com/partners/embo-press/editorial-policies#Peer%20review

** Click here to be directed to your login page: https://emboj.msubmit.net

EMBO Press Author Checklist

Corresponding Author Name: Johanne Jacobsen, Angelina Bilate	
Journal Submitted to: The EMBO Journal	
Manuscript Number: EMBOJ-2025-121127R	

USEFUL LINKS FOR COMPLETING THIS FORM
The EMBO Journal - Author Guidelines
EMBO Reports - Author Guidelines
Molecular Systems Biology - Author Guidelines
EMBO Molecular Medicine - Author Guidelines

Reporting Checklist for Life Science Articles (updated January
This checklist is adapted from Materials Design Analysis Reporting (MDAR) Checklist for Authors. MDAR establishes a minimum set of requirements in transparent reporting in the life sciences (see Statement of Task: 10.31222/osf.io/9sm4x). Please follow the journal's guidelines in preparing your
Please note that a copy of this checklist will be published alongside your article.

Abridged guidelines for figures
1. Data
The data shown in figures should satisfy the following conditions:
➞ the data were obtained and processed according to the field's best practice and are presented to reflect the results of the experiments in an accurate and unbiased manner.
➞ ideally, figure panels should include only measurements that are directly comparable to each other and obtained with the same assay.
➞ plots include clearly labeled error bars for independent experiments and sample sizes. Unless justified, error bars should not be shown for technical
➞ if n<5, the individual data points from each experiment should be plotted. Any statistical test employed should be justified.
➞ Source Data should be included to report the data underlying figures according to the guidelines set out in the authorship guidelines on Data

2. Captions
Each figure caption should contain the following information, for each panel where they are relevant:
➞ a specification of the experimental system investigated (eg cell line, species name).
➞ the assay(s) and method(s) used to carry out the reported observations and measurements.
➞ an explicit mention of the biological and chemical entity(ies) that are being measured.
➞ an explicit mention of the biological and chemical entity(ies) that are altered/varied/perturbed in a controlled manner.
➞ the exact sample size (n) for each experimental group/condition, given as a number, not a range;
➞ a description of the sample collection allowing the reader to understand whether the samples represent technical or biological replicates (including how many animals, litters, cultures, etc.).
➞ a statement of how many times the experiment shown was independently replicated in the laboratory.
➞ definitions of statistical methods and measures:
- common tests, such as t-test (please specify whether paired vs. unpaired), simple χ2 tests, Wilcoxon and Mann-Whitney tests, can be unambiguously identified by name only, but more complex techniques should be described in the methods section;
- are tests one-sided or two-sided?
- are there adjustments for multiple comparisons?
- exact statistical test results, e.g., P values = x but not P values < x;
- definition of 'center values' as median or average;
- definition of error bars as s.d. or s.e.m.

Please complete ALL of the questions below.
Select "Not Applicable" only when the requested information is not relevant for your study.

Materials

Newly Created Materials	Information included in the manuscript?	In which section is the information available? (Reagents and Tools Table, Materials and Methods, Figures, Data Availability Section)
New materials and reagents need to be available; do any restrictions apply?	Not Applicable	

Antibodies	Information included in the manuscript?	In which section is the information available? (Reagents and Tools Table, Materials and Methods, Figures, Data Availability Section)
For **antibodies** provide the following information:<br>- Commercial antibodies: RRID (if possible) or supplier name, catalogue number and or/clone number<br>- Non-commercial: RRID or citation	Yes	Reagents list

DNA and RNA sequences	Information included in the manuscript?	In which section is the information available? (Reagents and Tools Table, Materials and Methods, Figures, Data Availability Section)
**Short novel DNA or RNA including primers, probes**: provide the sequences.	Yes	Reagents list

Cell materials	Information included in the manuscript?	In which section is the information available? (Reagents and Tools Table, Materials and Methods, Figures, Data Availability Section)
**Cell lines:** Provide species information, strain. Provide accession number in repository **OR** supplier name, catalog number, clone number, and/**OR** RRID.	Yes	Methods and Reagents list
**Primary cultures:** Provide species, strain, sex of origin, genetic modification status.	Yes	Methods
Report if the cell lines were recently **authenticated** (e.g., by STR profiling) and tested for mycoplasma contamination.	Not Applicable	

Experimental animals	Information included in the manuscript?	In which section is the information available? (Reagents and Tools Table, Materials and Methods, Figures, Data Availability Section)
**Laboratory animals or Model organisms:** Provide species, strain, sex, age, genetic modification status. Provide accession number in repository **OR** supplier name, catalog number, clone number, **OR** RRID.	Yes	Methods
**Animal observed in or captured from the field:** Provide species, sex, and age where possible.	Not Applicable	
Please detail **housing and husbandry conditions**.	Yes	Methods

Plants and microbes	Information included in the manuscript?	In which section is the information available? (Reagents and Tools Table, Materials and Methods, Figures, Data Availability Section)
**Plants:** provide species and strain, ecotype and cultivar where relevant, unique accession number if available, and source (including location for collected wild specimens).	Not Applicable	
**Microbes:** provide species and strain, unique accession number if available, and source.	Not Applicable	

Human research participants	Information included in the manuscript?	In which section is the information available? (Reagents and Tools Table, Materials and Methods, Figures, Data Availability Section)
If collected and within the bounds of privacy constraints report on age, sex and gender or ethnicity for all study participants.	Not Applicable	

Core facilities	Information included in the manuscript?	In which section is the information available? (Reagents and Tools Table, Materials and Methods, Figures, Data Availability Section)
If your work benefited from core facilities, was their service mentioned in the acknowledgments section?	Yes	Acknowledgements

Design

Study protocol	Information included in the manuscript?	In which section is the information available? (Reagents and Tools Table, Materials and Methods, Figures, Data Availability Section)
If study protocol has been **pre-registered, provide DOI in the manuscript**. For clinical trials, provide the trial registration number **OR** cite DOI.	Not Applicable	
Report the **clinical trial registration number** (at ClinicalTrials.gov or equivalent), where applicable.	Not Applicable	

Laboratory protocol	Information included in the manuscript?	In which section is the information available? (Reagents and Tools Table, Materials and Methods, Figures, Data Availability Section)
Provide DOI OR other citation details if **external detailed step-by-step protocols** are available.	Not Applicable	

Experimental study design and statistics	Information included in the manuscript?	In which section is the information available? (Reagents and Tools Table, Materials and Methods, Figures, Data Availability Section)
Include a statement about **sample size** estimate even if no statistical methods were used.	Yes	Methods and Figure legends
Were any steps taken to minimize the effects of subjective bias when allocating animals/samples to treatment (e.g. **randomization procedure**)? If yes, have they been described?	Not Applicable	
Include a statement about **blinding** even if no blinding was done.	Yes	Methods and Figure legends
Describe **inclusion/exclusion criteria** if samples or animals were excluded from the analysis. Were the criteria pre-established?<br><br>If sample or data points were omitted from analysis, report if this was due to attrition or intentional exclusion and provide justification.	Not Applicable	
For every figure, are **statistical tests** justified as appropriate? Do the data meet the assumptions of the tests (e.g., normal distribution)? Describe any methods used to assess it. Is there an estimate of variation within each group of data? Is the variance similar between the groups that are being statistically compared?	Yes	Methods and Figure legends

Sample definition and in-laboratory replication	Information included in the manuscript?	In which section is the information available? (Reagents and Tools Table, Materials and Methods, Figures, Data Availability Section)
In the figure legends: state number of times the experiment was **replicated** in laboratory.	Yes	Figure legends
In the figure legends: define whether data describe **technical or biological replicates**.	Yes	Figure legends

Ethics

Ethics	Information included in the manuscript?	In which section is the information available? (Reagents and Tools Table, Materials and Methods, Figures, Data Availability Section)
Studies involving **human participants**: State details of **authority granting ethics approval** (IRB or equivalent committee(s), provide reference number for approval.	Not Applicable	
Studies involving **human participants**: Include a statement confirming that **informed consent** was obtained from all subjects and that the experiments conformed to the principles set out in the WMA Declaration of Helsinki and the Department of Health and Human Services Belmont Report.	Not Applicable	
Studies involving **human participants:** For publication of **patient photos**, include a statement confirming that consent to publish was obtained.	Not Applicable	
Studies involving experimental **animals**: State details of **authority granting ethics approval** (IRB or equivalent committee(s), provide reference number for approval. Include a statement of compliance with ethical regulations.	Yes	Methods
Studies involving **specimen and field samples:** State if relevant **permits** obtained, provide details of authority approving study; if none were required, explain why.	Not Applicable	

Dual Use Research of Concern (DURC)	Information included in the manuscript?	In which section is the information available? (Reagents and Tools Table, Materials and Methods, Figures, Data Availability Section)
Could your study fall under dual use research restrictions? Please check biosecurity documents and list of **select agents and toxins** (CDC): https://www.selectagents.gov/sat/list.htm	Not Applicable	
If you used a select agent, is the security level of the lab appropriate and reported in the manuscript?	Not Applicable	
If a study is subject to dual use research of concern regulations, is the name of the **authority granting approval and reference number** for the regulatory approval provided in the manuscript?	Not Applicable	

Reporting

The MDAR framework recommends adoption of discipline-specific guidelines, established and endorsed through community initiatives. Journals have their own policy about requiring specific guidelines and recommendations to complement MDAR.

Adherence to community standards	Information included in the manuscript?	In which section is the information available? (Reagents and Tools Table, Materials and Methods, Figures, Data Availability Section)
State if relevant guidelines or checklists (e.g., **ICMJE, MIBBI, ARRIVE, PRISMA**) have been followed or provided.	Not Applicable	
For **tumor marker prognostic studies,** we recommend that you follow the **REMARK** reporting guidelines (see link list at top right). See author guidelines, under 'Reporting Guidelines'. Please confirm you have followed these guidelines.	Not Applicable	
For **phase II and III randomized controlled trials**, please refer to the **CONSORT** flow diagram (see link list at top right) and submit the CONSORT checklist (see link list at top right) with your submission. See author guidelines, under 'Reporting Guidelines'. Please confirm you have submitted this list.	Not Applicable	

Data Availability

Data availability	Information included in the manuscript?	In which section is the information available? (Reagents and Tools Table, Materials and Methods, Figures, Data Availability Section)
Have **primary datasets** been deposited according to the journal's guidelines (see 'Data Deposition' section) and the respective accession numbers provided in the Data Availability Section?	Not Applicable	
Were **human clinical and genomic datasets** deposited in a public access-controlled repository in accordance to ethical obligations to the patients and to the applicable consent agreement?	Not Applicable	
Are **computational models** that are central and integral to a study available without restrictions in a machine-readable form? Were the relevant accession numbers or links provided?	Not Applicable	
If publicly available data were reused, provide the respective **data citations in the reference list**.	Not Applicable	
